# Inefficient Involvement of Insula in Sensorineural Hearing Loss

**DOI:** 10.3389/fnins.2019.00133

**Published:** 2019-02-20

**Authors:** Xiao-Min Xu, Yun Jiao, Tian-Yu Tang, Jian Zhang, Richard Salvi, Gao-Jun Teng

**Affiliations:** ^1^Jiangsu Key Laboratory of Molecular and Functional Imaging, Department of Radiology, Zhongda Hospital, Medical School of Southeast University, Nanjing, China; ^2^Center for Hearing and Deafness, University at Buffalo, Buffalo, NY, United States

**Keywords:** sensorineural hearing loss, insula, hyperperfusion, functional connectivity, cognition, emotion

## Abstract

The insular cortex plays an important role in multimodal sensory processing, audio-visual integration and emotion; however, little is known about how the insula is affected by auditory deprivation due to sensorineural hearing loss (SNHL). To address this issue, we used structural and functional magnetic resonance imaging to determine if the neural activity within the insula and its interregional functional connectivity (FC) was disrupted by SNHL and if these alterations were correlated clinical measures of emotion and cognition. Thirty-five SNHL subjects and 54 Controls enrolled in our study underwent auditory evaluation, neuropsychological assessments, functional and structure MRI, respectively. Twenty five patients and 20 Controls underwent arterial spin labeling scanning. FC of six insula subdivisions were assessed and the FC results were compared to the neuropsychological tests. Interregional connections were also compared among insula-associated networks, including salience network (SN), default mode network (DMN), and central executive network (CEN). Compared to Controls, SNHL subjects demonstrated hyperperfusion in the insula and significantly decreased FC between some insula subdivisions and other brain regions, including thalamus, putamen, precentral gyrus, postcentral gyrus, mid-cingulate cortex, dorsolateral prefrontal cortex, rolandic operculum. Anxiety, depression and cognitive impairments were correlated with FC values. Abnormal interactions among SN, DMN, and CEN were observed in SNHL group. Our result provides support for the “inefficient high-order control” theory of the insula in which the auditory deprivation caused by SNHL contributes to impaired sensory integration and central deficits in emotional and cognitive processing.

## Introduction

Sensorineural hearing loss (SNHL), primarily resulting from damage to the sensory hair cells and spiral ganglion neurons, arising from various etiologies, including neurodegenerative disease, noise, and ototoxic drugs (Cox et al., 2014; Wang et al., 2016; Crowson et al., 2017), is the most common sensory disorder affecting roughly one-eighth of the population (Edmiston and Mitchell, 2013; Cunningham and Tucci, 2017). An individual’s inability to hear and communicate effectively is associated with a broad range of non-auditory problems such as social isolation, depression, anxiety, and dementia resulting in a reduced quality of life (Bainbridge and Wallhagen, 2014; Basner et al., 2014; Henshaw et al., 2015; Kamil and Lin, 2015; Liu et al., 2016; Zhang et al., 2017a). The emotional and cognitive disorders associated with SNHL likely results from disturbances in neural networks outside the classical auditory pathway that integrate external and internal sensory information required for cognitive or emotional processes.

The insula, located deep within the lateral sulcus, has been implicated in numerous functions including emotion, awareness, cognition, motor control, and sensory processing (Craig, 2009; Bestelmeyer et al., 2014). Insula neurons have been found to respond directly to acoustic stimuli in a single-cell recording study (Bieser, 1998). Micarelli et al. (2017) highlighted hypoperfusion in the insula and auditory cortex in idiopathic sudden SNHL patients, reflecting a “freezing” behavior when auditory deprivation occurred abruptly. The function and structure of the insula were found to be impaired in patients with tinnitus and unilateral hearing loss using resting-state or task-based MRI approaches (Yang et al., 2014). Lesions of the insula or disconnections with the auditory cortex often result in auditory agnosia and musical anhedonia (Sihvonen et al., 2016). Damage to the insula can induce hyperacusis, a condition in which sounds are perceived as extremely loud (Boucher et al., 2015). Moreover, the insula has numerous connections with frontal cortex, cingulate cortex and amygdala which contribute to emotional, cognitive and other high level processes (Pandya et al., 1971; Hoistad and Barbas, 2008; Baur et al., 2013; Nomi et al., 2016), as well as neuropsychiatric disorders (Namkung et al., 2017), including depression, anxiety, and bipolar disorder (Stein et al., 2007; Liu et al., 2010; Hulvershorn et al., 2012). These preceding results suggest that the insula is a key structure of modulating acoustic information and could contribute to SNHL-associated psychiatric symptoms.

To elaborate the role of the insula in patients with SNHL, we first used voxel-based morphometry (VBM) to calculate the volume of the insula, then used arterial spin labeling (ASL) and fMRI to determine whether SNHL disrupted the activity and perfusion within the insula, altered the functional connectivity (FC) between the insula and other brain regions. We also examined the interactions among the salience network (SN), default mode network (DMN), and central executive network (CEN), due to the fact that the insula is a major component of SN, which plays an important role in switching CEN and DMN. Because of known regional differences (Deen et al., 2011), our analysis focused on six regions, the left and right ventral anterior insula (vAI), dorsal AI (dAI), and posterior insula (PI). To evaluate the potential significance of our finding, the functional changes in the insula associated with SNHL were correlated with clinical measures of neuropsychological function.

## Materials and Methods

### Participants

**FIGURE 1:**
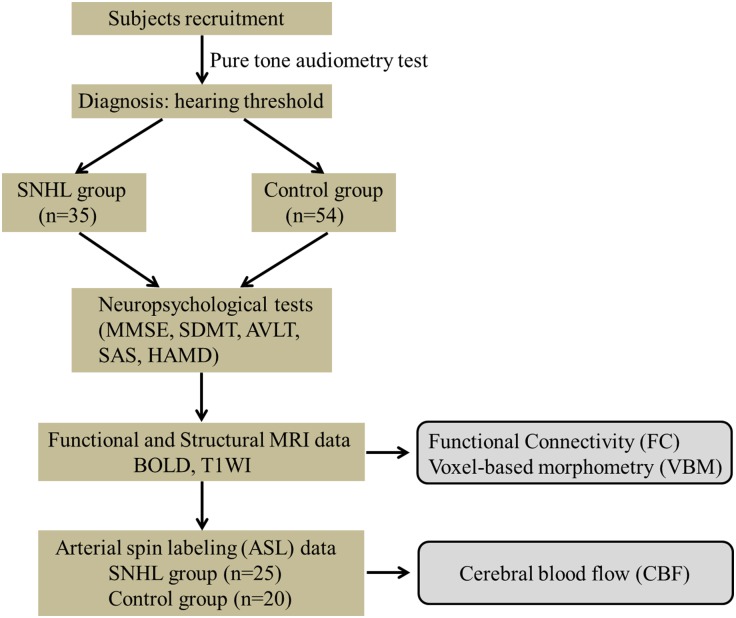
Experimental flowchart. SNHL, sensorineural hearing loss; MMSE, Mini Mental State Exam; SDMT, Symbol Digit Modalities Test; AVLT, Auditory Verbal Learning Test; SAS, Self-Rating Anxiety Scale; HAMD, Hamilton Depression Scale.

Eighty-nine participants were recruited from the ENT department of local hospitals or the local community for this study, 35 subjects with a mean age of 56.1 ± 8.6 years and a mean education of 10.9 ± 3.0 years had long-term bilateral SNHL and 54 subjects were age-, gender-, and education level-matched healthy controls with clinical normal hearing. Both groups underwent T1-weighted image scan (T1WI), BOLD sequences and a series of neuropsychological tests. Only 25 SNHL subjects (10 males and 15 females) and 20 Controls (4 males and 16 females) agreed to complete the next arterial spin labeling (ASL) scan (see Table 1 and Figure 1). All subjects were randomly assigned using double-blinded principles for further analysis.

**Table 1:** Clinical characteristics of SNHL and the control groups and local measurements of the left and right insula.

Characteristics	SNHL (*n* = 35)	Control (*n* = 54)	*P*-value
**Clinical measurements**
Gender (male/female)	22/13	34/20	0.992^a^
Age (years)	56.1 ± 8.6	53.5 ± 7.9	0.153^b^
Education (years)	10.9 ± 3.0	12.4 ± 5.2	0.136^b^
Duration (years)	6.9 ± 6.7	–	–
**Neuropsychological tests**
MMSE	29.6 ± 0.8	29.7 ± 0.5	0.352^b^
SDMT	33.4 ± 12.2	40.7 ± 8.9	0.003^b^
AVLT-5 min	5.7 ± 2.5	6.6 ± 2.0	0.070^b^
AVLT-20 min	5.6 ± 2.5	6.5 ± 2.2	0.079^b^
SAS	35.8 ± 7.1	30.9 ± 5.5	<0.001^b^
HAMD	6.1 ± 4.0	4.4 ± 2.9	0.025^b^
**Volume**
Relative volume of left insula	0.406 ± 0.007	0.411 ± 0.006	0.592^b^
Relative volume of right insula	0.405 ± 0.007	0.409 ± 0.006	0.612^b^
**Perfusion**	**SNHL (*n* = 25)**	**Control (*n* = 20)**	***P*-value**
Mean CBF of left insula (ml/100 g/min)	71.610 ± 2.847	61.100 ± 2.975	0.013^b^
Mean CBF of right insula (ml/100 g/min)	78.780 ± 3.109	67.230 ± 3.429	0.018^b^


*Data are represented as mean ± standard deviation. ^a^Chi-square test; ^b^Independent-sample *t*-test. SNHL, sensorineural hearing loss; MMSE, Mini Mental State Exam; SDMT, Symbol Digit Modalities Test; AVLT, Auditory Verbal Learning Test; SAS, Self-Rating Anxiety Scale; HAMD, Hamilton Depression Scale; CBF, cerebral blood flow*.

Participants were all right-handed and had at least 6 years of education, with ages ranging from 38 to 65 years. Patients meeting the diagnostic criteria of SNHL > 1 year. Participants were excluded if they (1) suffered from tinnitus, hyperacusis, acoustic neuroma, Meniere’s diseases, head trauma, visual loss, severe heart diseases or MRI contraindications; (2) had a history of head surgery, stroke, cognitive impairment or other serious physical and psychotic diseases; (3) presented with drug dependence, alcohol or substance abuse.

The study was approved by the ethics committee of Zhongda Hospital, Southeast University (2016ZDSYLL031.0). Written informed consent in accordance with the Declaration of Helsinki was obtained from all subjects.

### Auditory Evaluation

Clinical pure tone audiometry (PTA) test (Chen et al., 2018) was performed at 0.125, 0.25, 0.5, 1, 2, 4, 8 kHz by a trained otolaryngologist with 12 years of work experience from the ENT Department of Zhongda Hospital using a GSI-61 audiometer. In the control group, the mean PTA across the seven frequencies was <25 dB HL (Lin et al., 2011), while the mean PTA of SNHL across the seven frequencies was >30 dB HL. According to global burden of disease (GBD) 2013, hearing loss has been ranked into five severity levels, as moderate, 35–49 dB; moderately severe, 50–64 dB; severe, 65–79 dB; profound, 80–94 dB; and complete >95 dB.

### Neuropsychological Assessment

All subjects underwent a battery of neuropsychological tests prior to the MRI scans and these tests were carried out by a professional psychiatrist with 6 years of work experience to provide a measure of their cognitive and mental status. General cognition was established by the Mini Mental State Exam (MMSE) (Galea and Woodward, 2005), the Symbol Digit Modalities Test (SDMT) (Patel et al., 2017), and the Auditory Verbal Learning Test (AVLT) (Hawkins et al., 2004). Depression and anxiety status were assessed using the Hamilton Depression Scale (HAMD) (Maier et al., 1985) and the Self-Rating Anxiety Scale (SAS) (Zung, 1971).

### Brain Imaging Acquisition

MRI studies were performed in a Siemens 3.0 Tesla scanner using a homogeneous birdcage head coil. Subjects lay supine and were required to close their eyes, stay awake and avoid thinking specific thoughts while in the scanner. We used ear plugs and earphones to attenuate scanner noise and a head cushion to reduce head motion. High-resolution 3-dimensional T1WI scans were acquired using a spoiled gradient-echo sequence [repetition time (TR) = 1900 ms, echo time (TE) = 2.48 ms, flip angle (FA) = 90°, field of view (FOV) = 256 mm × 256 mm, acquisition matrix = 256 × 256, slices = 176, section thickness = 1.0 mm]. A gradient-echo-planar imaging sequence was set up to obtain functional images (TR = 2000 ms, TE = 25 ms, 36 slices, section thickness = 4.0 mm, FA = 90°, FOV = 240 mm × 240 mm, acquisition matrix = 64 × 64). Subsequent ASL perfusion MR was performed using a Siemens product pulsed-ASL (pASL) PICORE Q2T sequence (TR = 4000 ms, TE = 12 ms, 27 slices, thickness = 4 mm; FA = 90°; matrix = 64 × 64; FOV = 220 mm × 220 mm).

### Data Processing Protocol

#### Functional Data

Two experienced radiologists inspected all image data. As described in previous studies (Chen et al., 2014; Cui et al., 2014), data analysis was conducted using DPARBI toolbox^1^, which is based on DPARSF (version 4.3), SPM 12^2^, and REST^3^. After removing the first 10 time points, the remaining 230 times points were corrected for slice timing, realignment of head motion, segmentation and normalization to the non-linear Montreal Neurological Institute (MNI) template (resampling to 3 mm × 3 mm × 3 mm voxels). Then regressed six motion parameters, white matter, and CSF signals. Afterward, the images were smoothed using a 6 mm full-width half-maximum (FWHM) Gaussian kernel. No one was excluded from this study because of head motion >2.0 mm translation or >2.0° rotation in any direction.

#### Structure Data

Individual structural images were analyzed with the DARTEL-VBM method (Colloby et al., 2011, 2014) in the following order: (1) segment the MRI images into the gray matter (GM), white matter and cerebrospinal fluid standard unified segmentation model in SPM 12; (2) construct GM templates from the entire image dataset (Ashburner, 2007) to generate tissue probability maps in MNI space; (3) perform non-linear warping of segmented images to match the MNI space to DARTEL templates; (4) modulate the relative GM volume following spatial normalization; (5) smooth data with a 6 mm FWHM Gaussian kernel.

#### Perfusion Data

Similar to (Zhang et al., 2018), SPM 12 and ASLtbx (Wang et al., 2008) were used to process pASL data. First, motion corrections were performed to eliminate spurious motion artifacts. The raw ASL images were then high pass filtered to retain the higher frequency band. Then, the ASL images were co-registered to the T1 images and spatially smoothed with a 6 mm FWHM Gaussian kernel. After subsequent pairwise control/label image subtraction and cerebral blood flow (CBF) quantification, rejection of CBF outliers, mean CBF maps were created and registered into the MNI space using the transformation obtained from structural images.

### Statistical Analysis

Demographic, clinical variables and scores of neuropsychological performance were compared by independent-sample t-tests using SPSS software (Version 18.0, United States). Chi-square tests were used to compare categorical variables (e.g., gender). *P*-values <0.05 were considered statistically significant.

Using the Anatomical Automatic Labeling (AAL) atlas, we selected the left and right insula as two ROIs. We extracted the relative volume and mean CBF values of the two ROIs and identified changes in structure and perfusion caused by SNHL (two-sample *t*-test, *p* < 0.05). Consistent with previous research (Zamorano et al., 2017; Zhang et al., 2017b), bilateral vAI, dAI and PI subdivisions were used as seeds in the whole-brain FC analysis (see Table 2 for the coordinates of six ROIs). The resulting FC maps were transformed using Fisher’s z to yield normally distributed data and the data averaged for each subject. False discovery rate (FDR) correction was used to correct for multiple comparisons with a corrected *p* < 0.001. To examine changes in FC caused by SNHL, a partial correlation analysis with some covariates (including age, gender and education level) between *z*-values of the FC results and the clinical measurements were performed using SPSS software (Version 18.0, United States). Statistical significance was set a *p* < 0.05.

**Table 2:** Name and coordinates of insula subdivisions (*r* = 6 mm).

Hemisphere	Subdivision	Abbreviation	Coordinate		
			*X*	*Y*	*Z*
Left	Ventral anterior insula	vAI	-33	13	-7
	Dorsal anterior insula	dAI	-38	6	2
	Posterior insula	PI	-38	-6	5
Right	Ventral anterior insula	vAI	32	10	-6
	Dorsal anterior insula	dAI	35	7	3
	Posterior insula	PI	35	-11	6


The three large-scale networks, CEN, DMN, and SN, are believed to be involved in psychiatric disorders (Menon, 2011) and the insula is a key node of SN. Therefore, we investigated the changes in the interregional relations of these three networks following SNHL. The nodes representing CEN and DMN were selected according to previous research (Moran et al., 2013): CEN: dorsolateral prefrontal cortex (DLPFC), inferior parietal lobule (IPL), and Caudate; DMN: medial prefrontal cortex (MPFC), posterior cingulate cortex (PCC), lateral parietal cortex (LP), and parahippocampal gyrus (PHG). ROI-wise FC analysis was conducted among CEN, DMN, and SN, with FDR correction for multiple comparison (corrected *p* < 0.05). All above analysis on the MRI data were conducted using the age, gender and education level as covariance.

## Results

### Clinical Characteristics and Local Measurements of the Insula

**FIGURE 2:**
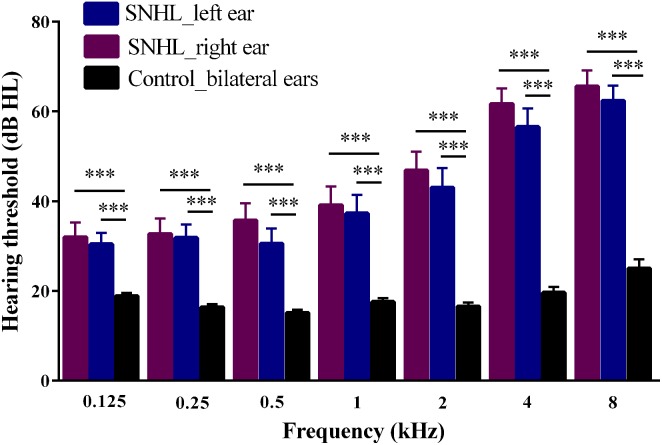
Hearing thresholds at seven frequencies (0.125, 0.25, 0.5, 1, 2, 4, 8 kHz) evaluated by pure tone audiometry tests in SNHL and Control groups. Data are expressed as mean ± standard error. ^∗∗∗^*p* < 0.001.

The demographic and clinical characteristics of SNHL and Controls are summarized in Table 1. Both groups were well-matched in terms of gender, age and education level. SNHL subjects performed significantly worse that Controls on SDMT (*p* < 0.003), SAS (*p* < 0.001) and HAMD (*p* = 0.025) tests. Figure 2 illustrates the differences in hearing thresholds between the SNHL group and Controls at 0.125, 0.25, 0.5, 1, 2, 4, and 8 kHz. Thresholds in the SNHL group were significantly higher in the SNHL group than Controls (*p* < 0.001), especially at the high frequencies (4 and 8 kHz).

There was no significant difference in the relative volume of bilateral insula between two groups (Table 1). However, CBF values of the left and right insula were significantly greater in SNHL group compared to Controls, indicating the hyperperfusion of the insula after auditory deprivation.

### Voxel-Wise Functional Connectivity of Insula Subdivisions

**FIGURE 3:**
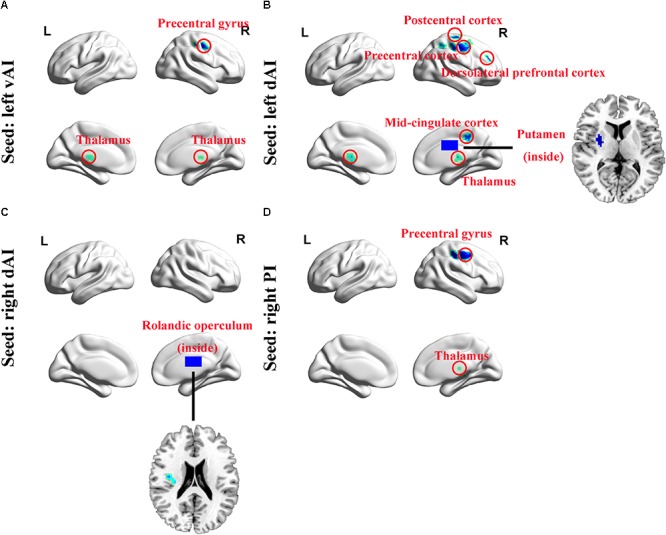
Whole-brain voxel-wise functional connectivity patterns of the insula subdivisions (*p* < 0.001, false discovery rate corrected). **(A)** Comparing with Controls, functional connectivity was significantly decreased between left vAI and bilateral thalamus and right precentral gyrus in SNHL subjects. **(B)** The left dAI showed significant decreased functional connectivity with right thalamus, putamen, mid-cingulate cortex, precentral gyrus, postcentral gyrus, and DLPFC. **(C)** The right dAI showed decreased connectivity with right rolandic operculum. **(D)** The right PI showed weakened connections with right thalamus and precentral gyrus. SNHL, sensorineural hearing loss; vAI, ventral anterior insula; dAI, dorsal anterior insula; PI, posterior insula; DLPFC, dorsolateral prefrontal cortex; L, left; R, right.

Using six insula subdivisions as ROIs, FC analysis revealed an extensive reduction of connectivity between the insula and other brain regions (*p* < 0.001, FDR corrected, minimum cluster = 50). Compared to Controls, the SNHL group showed a significant reduction in FC between the left vAI and the bilateral thalamus and right precentral gyrus. Additionally, the SNHL group displayed weakened FC between left dAI and the right thalamus, putamen, DLPFC, precentral gyrus, postcentral gyrus and mid-cingulate cortex. Moreover, the SNHL group demonstrated decreased FC between the right dAI and right rolandic operculum. Finally, the SNHL group demonstrated decreased FC between the right PI and right thalamus, as well as right precentral gyrus (Figure 3 and Table 3).

**Table 3:** Decreased functional connectivity of the insula subdivisions in SNHL subjects comparing with Controls.

Brain region	BA	Side	MNI coordinate			Peak *t*-value	Cluster size
			***X***	***Y***	***Z***		
**Functional connectivity of left vAI**
Thalamus	–	R	12	-22	9	-7.2482	102
Thalamus	–	L	-6	-18	6	-6.736	57
Precentral gyrus	6	R	42	-12	45	-6.3206	68
**Functional connectivity of left dAI**
Thalamus	–	R	6	-21	9	-7.6285	298
Putamen	48	R	30	-3	9	-5.8026	81
Dorsolateral prefrontal cortex	46	R	42	42	27	-5.3125	53
Precentral gyrus	6	R	42	-12	45	-6.227	303
Mid-cingulate cortex	–	R	15	-36	39	-5.522	53
Postcentral gyrus	6	R	27	-27	66	-5.3037	62
**Functional connectivity of right dAI**
Rolandic operculum	48	R	39	-12	21	-5.8744	56
**Functional connectivity of right PI**
Thalamus	–	L	9	-21	9	-6.4883	108
Precentral gyrus	6	R	39	-12	45	-6.4082	229


*The threshold was set at *p* < 0.001, false discovery rate correction. BA, Brodmann area; vAI, ventral anterior insula; dAI, dorsal anterior insula; PI, posterior insula.*

### Functional Connectivity and Clinical Features

**FIGURE 4:**
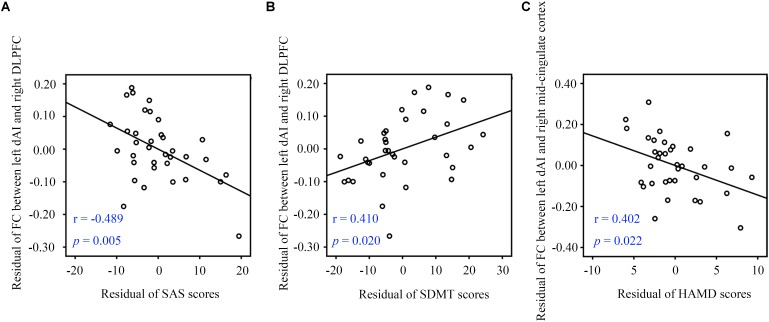
Partial correlations between fMRI data and clinical measurements. **(A)** The left dAI-right DLPFC connectivity showed a negative correlation with SAS scores. **(B)** The left dAI-right DLPFC connectivity showed a positive correlation with SDMT scores. **(C)** The left dAI-right mid-cingulate cortex connectivity showed a negative correlation with HAMD scores. SNHL, sensorineural hearing loss; dAI, dorsal anterior insula; SDMT, Symbol Digit Modalities Test; SAS, Self-Rating Anxiety Scale; HAMD, Hamilton Depression Scale; DLPFC, dorsolateral prefrontal cortex.

Partial correlation analysis with some covariates (age, gender, and education level) identified significant relationships between clinical characteristics and FC data. In the SNHL group, FC of the left dAI and right DLPFC was negatively correlated with SAS scores (*r* = -0.489, *p* = 0.005, Figure 4A) and positively correlated with SDMT sores (*r* = 0.410, *p* = 0.020, Figure 4B). FC in the left dAI and the right mid-cingulate cortex FC was negatively correlated with HAMD scores (*r* = -0.402, *p* = 0.022, Figure 4C). However, no correlation survived after multiple comparison.

### Network Abnormalities in CEN, DMN, and SN

**FIGURE 5:**
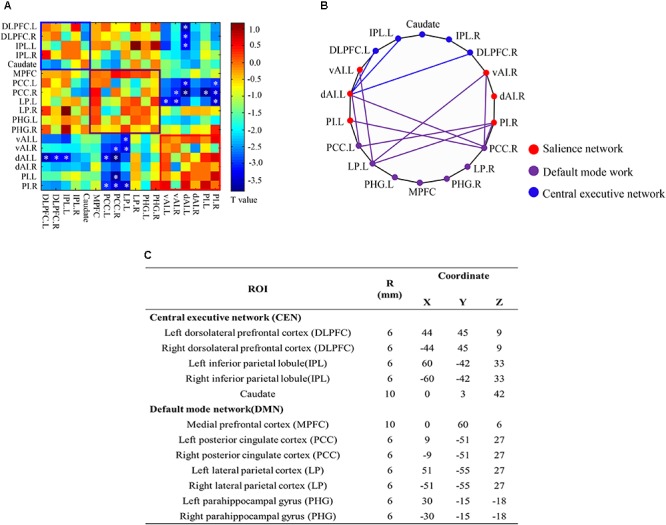
ROI-wise interregional connectivity analysis among salience network, central executive network and default mode network across all participants (*p* < 0.05, false discovery rate corrected). **(A)** Colored matrix indicating *t*-values of interactions among salience network, central executive network and default mode network. **(B)** Significant difference in interregional functional connectivity between SNHL and Controls. **(C)** Detailed ROI coordinates extracted from central executive network and default mode network. SNHL, sensorineural hearing loss. ^∗^*p* < 0.05.

To determine if SNHL affected the 18 core regions involved in the CEN, DMN, and SN, we employed a ROI-wise interregional connectivity analysis to identify significant changes (Figure 5). SNHL significantly weakened interactions among these three networks as follows. The left vAI showed decreased interregional connectivity with the left LP while the right vAI showed decreased connections with left LP and right PCC. And the left dAI showed reduced connectivity with bilateral DLPFC, PCC and right PCC. Meanwhile, SNHL reduced the connectivity between left PI and right PCC, as well as the connections between right PI and bilateral PCC and left LP (*p* < 0.05, FDR corrected).

## Discussion

To our knowledge, this is the first study to determine if SNHL disrupts the function and connective of the insula and interregional connectivity using a multimodal neuroimaging approach. We found significant hyperperfusion in left and right insula in subjects with SNHL and widespread decreased neuronal synchronizations between insula subdivisions and other brain regions. Several of these FC changes were correlated with altered performance on neuropsychological metrics of emotion and cognition. Furthermore, SNHL altered the interregional connections among CEN, DMN, and SN. Taken together, our findings provide new insights on the role of insula in SNHL-related neuropsychiatric deficits.

### SNHL and Structure

Our VBM analysis failed to identify significant volume differences in the insula between groups, consistent with absence of major structural changes in the insula of prelingual and postlingual hearing loss (Shibata, 2007; Hribar et al., 2014; Shiell and Zatorre, 2017). However, a few studies (Allen et al., 2008) reported structural alterations in the human insula from congenital deafness; in these cases sign language increased GM volume of left PI compared to normal hearing subjects. Compared to hearing non-signers, hearing signers, and deaf signers exhibited a significant increase in the size of the right insula, which was attributed to lip-reading and articulation, rather than auditory input itself (MacSweeney et al., 2001). In contrast, the SNHL patients in our study all had acquired postlingual hearing loss and lived with normal hearing families. Thus, the most likely explanation for the lack of structural changes in our study was the lack of sign language history. We did not ask our controls and SNHL whether they were signers and cannot address the question about the effects of articulation. All things being equal, it is likely that signing was equally distributed across the SNHL and the control group, making it difficult to identify an articulatory effect in our data.

### Perfusion

Pulsed-ASL is a non-invasive and sensitive imaging technique for assessing CBF without using radioactive sources or contrast agents (Williams et al., 1992; Detre and Alsop, 1999). In comparison to PET, pASL has superior spatial resolution and sensitivity, therefore it has been widely used to measure CBF in cerebrovascular and psychiatric disorders (Cui et al., 2017). Our observation of heighted CBF values in the insula was independent of cortical atrophy, as no volume difference were observed between SNHL subjects and Controls in the insula. Lee et al. (2001) reported hypoperfusion in the primary cortex and auditory-association cortex of prelingually deaf patients; the hypoperfusion was positively correlated with degree of hearing improvement after cochlear implantation. Prior PET reports from subject with postlingual deafness (*n* = 7) (Okuda et al., 2013) and idiopathic sudden SNHL patients (*n* = 14) (Micarelli et al., 2017) indicated lower glucose metabolism in the temporal lobe and insula attributed to the absence of sensory input. The difference between our insula results and these earlier reports may be related to the types of hearing loss (i.e., unilateral vs. bilateral, genetic factors), duration of deafness, small sample size, and other factors such as cognitive and emotional differences as illustrated by worse scores on SDMT, SAS, and HAMD in our SNHL subjects. Others have noted a relationship between the perfusion of the insula and anxiety (Andreescu et al., 2011). Moreover, it has been reported that depression could activate the insula and amygdala, propounding an “anxious-misery” dimension (Kennedy et al., 2001; Xekardaki et al., 2015). Thus, the increased perfusion in the insula might be associated with SNHL-induced anxiety and depression in our subject sample. We speculate that the CBF could be a confirmatory candidate biomarker for diagnosis since it is tightly linked to regional metabolism and neural activity.

### Regional Insula Functional Connectivity Changes

Most insula subdivisions in our SNHL subjects showed decreased connectivity with the thalamus which might reflect a change in sensory-emotional integration (Groenewegen and Berendse, 1994). The thalamus receives numerous sensory inputs and then relays this information to associated higher-order primary cortical areas (Lee, 2015). Reward stimuli can increase the thalamus-to-insula connectivity (Cho et al., 2013) whereas negative stimuli, such as difficulties hearing due to partial deafness, could have the opposite effect thereby reducing connectivity. It is conceivable that reduced incoming auditory information results in weakened connectivity between the thalamus and insula. Additionally, we observed reduced connections between the left dAI and right putamen while the putamen has been found to connect to the thalamus and functioned in cognition decline, anxiety and depression (Park et al., 2017; Luo et al., 2018).

Traditionally, the dAI and vAI are activated by cognition and emotion task, respectively (Nomi et al., 2016). However, this cognition-emotion dichotomy has been challenged recently (Uddin et al., 2014). Our data showed reduced couplings between the left dAI and right mid-cingulate cortex in SNHL subjects, as well as the negative correlation with HAMD scores. The mid-cingulate cortex is not only linked to cognitive processing, including decision making, attention and salience (Dosenbach et al., 2007; Rushworth et al., 2007), but also homeostasis and emotion, and it has become a therapy target for intractable mood, anxiety, and pain disorders (Tolomeo et al., 2016). Patients with treatment-resistant depression or refractory pain sometimes receive bilateral cingulotomy (including AI) (Steele et al., 2008; Shackman et al., 2011). Primate and human studies (Mufson and Mesulam, 1982; Taylor et al., 2009) provide evidence for functional connections between the AI and mid-cingulate cortex, as well as FC between the entire insula and mid-cingulate cortex. In addition, thalamus-insula- mid-cingulate cortex projection tracts have been found in animals (Hatanaka et al., 2003), suggesting that functional connections between the dAI and mid-cingulate cortex might contribute to neuropsychiatric disorders.

Reduced fluorodeoxyglucose (FDG) uptake has been observed in the insula, precentral gyrus and postcentral gyrus within 72 h following sudden SNHL (Micarelli et al., 2017). Although the postcentral gyrus has often been linked to somatosensory processing, pain and olfaction (Bedny et al., 2011; Grabski et al., 2012), studies have demonstrated greater activation in this region in speech rhythm, auditory oddball and articulation imagery tasks (Geiser et al., 2008; Job et al., 2012; Tian et al., 2016), suggesting its involvement in speech perception. The volume of the precentral gyrus is reduced in schizophrenia (Zhou et al., 2005), indicating that the precentral gyrus (part of the primary motor cortex) is involved in motor-related cognitive functioning (Georgopoulos, 2000); these observations are relevant to poorer SDMT performance in our SNHL subjects. Moreover, the right dAI showed decreased connectivity with rolandic operculum as it together covers with the insula and was reported hyperactivity in tinnitus subjects following acute acoustic trauma (Job et al., 2012).

Some insula subdivisions showed decreased connectivity with the DLPFC, PCC, and LP using voxel-wise and ROI-wise methods, suggesting the involvement of the DMN and CEN in SNHL-induced auditory deprivation. A meta-analysis showed that several brain regions overlap with the DMN and CEN play an important role in processing multiple cognitive signals (Dosenbach et al., 2006), while acquiring normal sensory information supports cognition functioning. SNHL disrupts the auditory system, leading to the disruptions of the SN, resulting in imbalances between the DMN and CEN. Chand et al. (2017), also found abnormal interactions among the SN, DMN, and CEN in subjects with mild cognitive impairments. Recent research suggest that that depression and anxiety are associated with alterations in the DMN, expanding the role of DMN in emotion processing (Vicentini et al., 2017), as FC of left dAI-right DLPFC not only showed positive correlation with SDMT performance, but also negative correlation with SAS scores. Combing the hyperperfusion in the insula, we used an “inefficient high-order control” theory to illustrate this phenomenon.

### Limitation

There were several limitations to our research. First, it is reported that human left and right insula occupy a total volume of around 10–20 cm^3^ (Bauernfeind et al., 2013) while we employed 6-mm diameter spherical ROIs as seeds, further research with independent component analysis could be used to extract the insula subregions. Second, the relative small sample of subjects likely reduced the statistical power, particularly for the correlation analysis. A follow-up study with a larger dataset might help to elucidate more subtle effects in the future. Another limitation was the range and duration of hearing loss in our study. Repeating aspects of this study using subjects with more severe and/or longer duration hearing loss might identify other functional and structural changes associated with SNHL.

## Conclusion

In conclusion, this study underscores the potential contribution of disrupted neural processing in the insula resulting from SNHL and its potential relationships with neuropsychiatric disorders, suggesting that it could be a candidate biomarker for auditory deprivation related neural deficits and a future target for therapy.

## Author Contributions

X-MX collected the fMRI data, performed the analysis, and wrote the manuscript. YJ and T-YT contributed to fMRI data analysis and discussion. JZ helped with data collection. RS reviewed and revised the manuscript. G-JT designed the MRI experiments and contributed to the manuscript revision.

## Conflict of Interest Statement

The authors declare that the research was conducted in the absence of any commercial or financial relationships that could be construed as a potential conflict of interest.

## Footnotes

1 http://rfmri.org/DPABI

2 http://www.fil.ion.ucl.ac.uk/spm

3 http://www.restfmri.net

## References

[B1] Allen J. S. Emmorey K. Bruss J. Damasio H. (2008). Morphology of the insula in relation to hearing status and sign language experience. *J. Neurosci.* 28 11900–11905. 10.1523/JNEUROSCI.3141-08.2008 19005055 PMC2606108

[B2] Andreescu C. Gross J. J. Lenze E. Edelman K. D. Snyder S. Tanase C. (2011). Altered cerebral blood flow patterns associated with pathologic worry in the elderly. *Depress. Anxiety* 28 202–209. 10.1002/da.20799 21394853 PMC3225118

[B3] Ashburner J. (2007). A fast diffeomorphic image registration algorithm. *Neuroimage* 38 95–113. 10.1016/j.neuroimage.2007.07.007 17761438

[B4] Bainbridge K. E. Wallhagen M. I. (2014). Hearing loss in an aging American population: extent, impact, and management. *Annu. Rev. Public Health* 35 139–152. 10.1146/annurev-publhealth-032013-182510 24641557

[B5] Basner M. Babisch W. Davis A. Brink M. Clark C. Janssen S. (2014). Auditory and non-auditory effects of noise on health. *Lancet* 383 1325–1332. 10.1016/S0140-6736(13)61613-X. 24183105 PMC3988259

[B6] Bauernfeind A. L. de Sousa A. A. Avasthi T. Dobson S. D. Raghanti M. A. Lewandowski A. H. (2013). A volumetric comparison of the insular cortex and its subregions in primates. *J. Hum. Evol.* 64 263–279. 10.1016/j.jhevol.2012.12.003 23466178 PMC3756831

[B7] Baur V. Hanggi J. Langer N. Jancke L. (2013). Resting-state functional and structural connectivity within an insula-amygdala route specifically index state and trait anxiety. *Biol. Psychiatry* 73 85–92. 10.1016/j.biopsych.2012.06.003 22770651

[B8] Bedny M. Pascual-Leone A. Dodell-Feder D. Fedorenko E. Saxe R. (2011). Language processing in the occipital cortex of congenitally blind adults. *Proc. Natl. Acad. Sci. U.S.A.* 108 4429–4434. 10.1073/pnas.1014818108 21368161 PMC3060248

[B9] Bestelmeyer P. E. Maurage P. Rouger J. Latinus M. Belin P. (2014). Adaptation to vocal expressions reveals multistep perception of auditory emotion. *J. Neurosci.* 34 8098–8105. 10.1523/JNEUROSCI.4820-13.2014 24920615 PMC4051968

[B10] Bieser A. (1998). Processing of twitter-call fundamental frequencies in insula and auditory cortex of squirrel monkeys. *Exp. Brain Res.* 122 139–148. 10.1007/s002210050501 9776512

[B11] Boucher O. Turgeon C. Champoux S. Menard L. Rouleau I. Lassonde M. (2015). Hyperacusis following unilateral damage to the insular cortex: a three-case report. *Brain Res.* 1606 102–112. 10.1016/j.brainres.2015.02.030 25721796

[B12] Chand G. B. Wu J. Hajjar I. Qiu D. (2017). Interactions of the salience network and its subsystems with the default-mode and the central-executive networks in normal aging and mild cognitive impairment. *Brain Connect.* 7 401–412. 10.1089/brain.2017.0509 28707959 PMC5647507

[B13] Chen Y. C. Chen H. Jiang L. Bo F. Xu J. J. Mao C. N. (2018). Presbycusis disrupts spontaneous activity revealed by resting-state functional MRI. *Front. Behav. Neurosci.* 12:44. 10.3389/fnbeh.2018.00044 29593512 PMC5859072

[B14] Chen Y. C. Jiao Y. Cui Y. Shang S. A. Ding J. Feng Y. (2014). Aberrant brain functional connectivity related to insulin resistance in type 2 diabetes: a resting-state fMRI study. *Diabetes Care* 37 1689–1696. 10.2337/dc13-2127 24658392

[B15] Cho Y. T. Fromm S. Guyer A. E. Detloff A. Pine D. S. Fudge J. L. (2013). Nucleus accumbens, thalamus and insula connectivity during incentive anticipation in typical adults and adolescents. *Neuroimage* 66 508–521. 10.1016/j.neuroimage.2012.10.013 23069809 PMC3949208

[B16] Colloby S. J. Firbank M. J. Vasudev A. Parry S. W. Thomas A. J. O’Brien J. T. (2011). Cortical thickness and VBM-DARTEL in late-life depression. *J. Affect. Disord.* 133 158–164. 10.1016/j.jad.2011.04.010 21550668

[B17] Colloby S. J. O’Brien J. T. Taylor J. P. (2014). Patterns of cerebellar volume loss in dementia with Lewy bodies and Alzheimer’s disease: a VBM-DARTEL study. *Psychiatry Res. Neuroimag.* 223 187–191. 10.1016/j.pscychresns.2014.06.006 25037902 PMC4333903

[B18] Cox R. M. Johnson J. A. Xu J. (2014). Impact of advanced hearing aid technology on speech understanding for older listeners with mild to moderate, adult-onset, sensorineural hearing loss. *Gerontology* 60 557–568. 10.1159/000362547 25139516 PMC4224118

[B19] Craig A. D. (2009). How do you feel - now? The anterior insula and human awareness. *Nat. Rev. Neurosci.* 10 59–70. 10.1038/nrn2555 19096369

[B20] Crowson M. G. Hertzano R. Tucci D. L. (2017). Emerging therapies for sensorineural hearing loss. *Otol. Neurotol.* 38 792–803. 10.1097/MAO.0000000000001427 28383465 PMC5465007

[B21] Cui Y. Jiao Y. Chen Y. C. Wang K. Gao B. Wen S. (2014). Altered spontaneous brain activity in type 2 diabetes: a resting-state functional MRI study. *Diabetes* 63 749–760. 10.2337/db13-0519 24353185

[B22] Cui Y. Liang X. Gu H. Hu Y. Zhao Z. Yang X. Y. (2017). Cerebral perfusion alterations in type 2 diabetes and its relation to insulin resistance and cognitive dysfunction. *Brain Imaging Behav.* 11 1248–1257. 10.1007/s11682-016-9583-9 27714551 PMC5653700

[B23] Cunningham L. L. Tucci D. L. (2017). Hearing loss in adults. *N. Engl. J. Med.* 377 2465–2473. 10.1056/NEJMra1616601 29262274 PMC6457651

[B24] Deen B. Pitskel N. B. Pelphrey K. A. (2011). Three systems of insular functional connectivity identified with cluster analysis. *Cereb. Cortex* 21 1498–1506. 10.1093/cercor/bhq186 21097516 PMC3116731

[B25] Detre J. A. Alsop D. C. (1999). Perfusion magnetic resonance imaging with continuous arterial spin labeling: methods and clinical applications in the central nervous system. *Eur. J. Radiol.* 30 115–124. 10.1016/S0720-048X(99)00050-9 10401592

[B26] Dosenbach N. U. Fair D. A. Miezin F. M. Cohen A. L. Wenger K. K. Dosenbach R. A. (2007). Distinct brain networks for adaptive and stable task control in humans. *Proc. Natl. Acad. Sci. U.S.A.* 104 11073–11078. 10.1073/pnas.0704320104 17576922 PMC1904171

[B27] Dosenbach N. U. Visscher K. M. Palmer E. D. Miezin F. M. Wenger K. K. Kang H. C. (2006). A core system for the implementation of task sets. *Neuron* 50 799–812. 10.1016/j.neuron.2006.04.031 16731517 PMC3621133

[B28] Edmiston R. Mitchell C. (2013). Hearing loss in adults. *BMJ* 346:f2496. 10.1136/bmj.f2496. 23618723

[B29] Galea M. Woodward M. (2005). Mini-Mental State Examination (MMSE). *Aust. J. Physiother.* 51:198 10.1016/S0004-9514(05)70034-9 16187459

[B30] Geiser E. Zaehle T. Jancke L. Meyer M. (2008). The neural correlate of speech rhythm as evidenced by metrical speech processing. *J. Cogn. Neurosci.* 20 541–552. 10.1162/jocn.2008.20029 18004944

[B31] Georgopoulos A. P. (2000). Neural aspects of cognitive motor control. *Curr. Opin. Neurobiol.* 10 238–241. 10.1016/S0959-4388(00)00072-6 10753794

[B32] Grabski K. Lamalle L. Sato M. (2012). Somatosensory-motor adaptation of orofacial actions in posterior parietal and ventral premotor cortices. *PLoS One* 7:e49117. 10.1371/journal.pone.0049117 23185300 PMC3502466

[B33] Groenewegen H. J. Berendse H. W. (1994). The specificity of the nonspecific midline and intralaminar thalamic nuclei. *Trends Neurosci.* 17 52–57 10.1016/0166-2236(94)90074-4 7512768

[B34] Hatanaka N. Tokuno H. Hamada I. Inase M. Ito Y. Imanishi M. (2003). Thalamocortical and intracortical connections of monkey cingulate motor areas. *J. Comp. Neurol.* 462 121–138. 10.1002/cne.10720 12761828

[B35] Hawkins K. A. Dean D. Pearlson G. D. (2004). Alternative forms of the rey auditory verbal learning test: a review. *Behav. Neurol.* 15 99–107. 10.1155/2004/940191 15706053 PMC5488610

[B36] Henshaw H. Sharkey L. Crowe D. Ferguson M. (2015). Research priorities for mild-to-moderate hearing loss in adults. *Lancet* 386 2140–2141. 10.1016/S0140-6736(15)01048-X 26638958

[B37] Hoistad M. Barbas H. (2008). Sequence of information processing for emotions through pathways linking temporal and insular cortices with the amygdala. *Neuroimage* 40 1016–1033. 10.1016/j.neuroimage.2007.12.043 18261932 PMC2680198

[B38] Hribar M. Suput D. Carvalho A. A. Battelino S. Vovk A. (2014). Structural alterations of brain grey and white matter in early deaf adults. *Hear Res.* 318 1–10. 10.1016/j.heares.2014.09.008 25262621

[B39] Hulvershorn L. A. Karne H. Gunn A. D. Hartwick S. L. Wang Y. Hummer T. A. (2012). Neural activation during facial emotion processing in unmedicated bipolar depression, euthymia, and mania. *Biol. Psychiatry* 71 603–610. 10.1016/j.biopsych.2011.10.038 22206876 PMC3703667

[B40] Job A. Pons Y. Lamalle L. Jaillard A. Buck K. Segebarth C. (2012). Abnormal cortical sensorimotor activity during “Target” sound detection in subjects with acute acoustic trauma sequelae: an fMRI study. *Brain Behav.* 2 187–199. 10.1002/brb3.21 22574285 PMC3345361

[B41] Kamil R. J. Lin F. R. (2015). The effects of hearing impairment in older adults on communication partners: a systematic review. *J. Am. Acad. Audiol.* 26 155–182. 10.3766/jaaa.26.2.6 25690776

[B42] Kennedy B. L. Schwab J. J. Morris R. L. Beldia G. (2001). Assessment of state and trait anxiety in subjects with anxiety and depressive disorders. *Psychiatr. Q.* 72 263–276. 10.1023/A:1010305200087 11467160

[B43] Lee C. C. (2015). Exploring functions for the non-lemniscal auditory thalamus. *Front. Neural Cir.* 9:69. 10.3389/fncir.2015.00069 26582978 PMC4631820

[B44] Lee D. S. Lee J. S. Oh S. H. Kim S. K. Kim J. W. Chung J. K. (2001). Cross-modal plasticity and cochlear implants. *Nature* 409 149–150. 10.1038/35051653 11196628

[B45] Lin F. R. Thorpe R. Gordon-Salant S. Ferrucci L. (2011). Hearing loss prevalence and risk factors among older adults in the United States. *J. Gerontol. A Biol. Sci. Med. Sci.* 66 582–590. 10.1093/gerona/glr002 21357188 PMC3074958

[B46] Liu L. Shen P. He T. Chang Y. Shi L. Tao S. (2016). Noise induced hearing loss impairs spatial learning/memory and hippocampal neurogenesis in mice. *Sci. Rep.* 6:20374. 10.1038/srep20374 26842803 PMC4740884

[B47] Liu Z. Xu C. Xu Y. Wang Y. Zhao B. Lv Y. (2010). Decreased regional homogeneity in insula and cerebellum: a resting-state fMRI study in patients with major depression and subjects at high risk for major depression. *Psychiatry Res.* 182 211–215. 10.1016/j.pscychresns.2010.03.004 20493670

[B48] Luo L. Becker B. Zheng X. Zhao Z. Xu X. Zhou F. (2018). A dimensional approach to determine common and specific neurofunctional markers for depression and social anxiety during emotional face processing. *Hum. Brain Mapp.* 39 758–771. 10.1002/hbm.23880 29105895 PMC6866417

[B49] MacSweeney M. Campbell R. Calvert G. A. McGuire P. K. David A. S. Suckling J. (2001). Dispersed activation in the left temporal cortex for speech-reading in congenitally deaf people. *Proc. Biol. Sci.* 268 451–457. 10.1098/rspb.2000.0393 11296856 PMC1088627

[B50] Maier W. Philipp M. Gerken A. (1985). [Dimensions of the Hamilton Depression Scale. Factor analysis studies]. *Eur. Arch. Psychiatry Neurol. Sci.* 234 417–422. 10.1007/BF00386061 4029226

[B51] Menon V. (2011). Large-scale brain networks and psychopathology: a unifying triple network model. *Trends Cogn. Sci.* 15 483–506. 10.1016/j.tics.2011.08.003 21908230

[B52] Micarelli A. Chiaravalloti A. Viziano A. Danieli R. Schillaci O. Alessandrini M. (2017). Early cortical metabolic rearrangement related to clinical data in idiopathic sudden sensorineural hearing loss. *Hear Res.* 350 91–99. 10.1016/j.heares.2017.04.011 28460253

[B53] Moran L. V. Tagamets M. A. Sampath H. O’Donnell A. Stein E. A. Kochunov P. (2013). Disruption of anterior insula modulation of large-scale brain networks in schizophrenia. *Biol. Psychiatry* 74 467–474. 10.1016/j.biopsych.2013.02.029 23623456 PMC3735654

[B54] Mufson E. J. Mesulam M. M. (1982). Insula of the old world monkey. II: afferent cortical input and comments on the claustrum. *J. Comp. Neurol.* 212 23–37. 10.1002/cne.902120103 7174906

[B55] Namkung H. Kim S. Sawa A. (2017). The insula: an underestimated brain area in clinical neuroscience, psychiatry, and neurology. *Trends Neurosci.* 40 200–207. 10.1016/j.tins.2017.02.002 28314446 PMC5538352

[B56] Nomi J. S. Farrant K. Damaraju E. Rachakonda S. Calhoun V. D. Uddin L. Q. (2016). Dynamic functional network connectivity reveals unique and overlapping profiles of insula subdivisions. *Hum. Brain Mapp.* 37 1770–1787. 10.1002/hbm.23135 26880689 PMC4837017

[B57] Okuda T. Nagamachi S. Ushisako Y. Tono T. (2013). Glucose metabolism in the primary auditory cortex of postlingually deaf patients: an FDG-PET study. *ORL J. Otorhinolaryngol. Relat. Spec.* 75 342–349. 10.1159/000357474 24435067

[B58] Pandya D. N. Karol E. A. Heilbronn D. (1971). The topographical distribution of interhemispheric projections in the corpus callosum of the rhesus monkey. *Brain Res.* 32 31–43. 10.1016/0006-8993(71)90153-3 5000193

[B59] Park I. H. Lee B. C. Kim J. J. Kim J. I. Koo M. S. (2017). Effort-based reinforcement processing and functional connectivity underlying amotivation in medicated patients with depression and schizophrenia. *J. Neurosci.* 37 4370–4380. 10.1523/JNEUROSCI.2524-16.2017 28283562 PMC6596561

[B60] Patel V. P. Walker L. A. S. Feinstein A. (2017). Deconstructing the symbol digit modalities test in multiple sclerosis: the role of memory. *Mult. Scler. Relat. Disord.* 17 184–189. 10.1016/j.msard.2017.08.006 29055455

[B61] Rushworth M. F. Buckley M. J. Behrens T. E. Walton M. E. Bannerman D. M. (2007). Functional organization of the medial frontal cortex. *Curr. Opin. Neurobiol.* 17 220–227. 10.1016/j.conb.2007.03.001 17350820

[B62] Shackman A. J. Salomons T. V. Slagter H. A. Fox A. S. Winter J. J. Davidson R. J. (2011). The integration of negative affect, pain and cognitive control in the cingulate cortex. *Nat. Rev. Neurosci.* 12 154–167. 10.1038/nrn2994 21331082 PMC3044650

[B63] Shibata D. K. (2007). Differences in brain structure in deaf persons on MR imaging studied with voxel-based morphometry. *AJNR Am. J. Neuroradiol.* 28 243–249. 17296987 PMC7977390

[B64] Shiell M. M. Zatorre R. J. (2017). White matter structure in the right planum temporale region correlates with visual motion detection thresholds in deaf people. *Hear Res.* 343 64–71. 10.1016/j.heares.2016.06.011 27321204

[B65] Sihvonen A. J. Ripolles P. Leo V. Rodriguez-Fornells A. Soinila S. Sarkamo T. (2016). Neural basis of acquired amusia and its recovery after stroke. *J. Neurosci.* 36 8872–8881. 10.1523/JNEUROSCI.0709-16.2016 27559169 PMC6601900

[B66] Steele J. D. Christmas D. Eljamel M. S. Matthews K. (2008). Anterior cingulotomy for major depression: clinical outcome and relationship to lesion characteristics. *Biol. Psychiatry* 63 670–677. 10.1016/j.biopsych.2007.07.019 17916331

[B67] Stein M. B. Simmons A. N. Feinstein J. S. Paulus M. P. (2007). Increased amygdala and insula activation during emotion processing in anxiety-prone subjects. *Am. J. Psychiatry* 164 318–327. 10.1176/ajp.2007.164.2.318 17267796

[B68] Taylor K. S. Seminowicz D. A. Davis K. D. (2009). Two systems of resting state connectivity between the insula and cingulate cortex. *Human Brain Mapp.* 30 2731–2745. 10.1002/hbm.20705 19072897 PMC6871122

[B69] Tian X. Zarate J. M. Poeppel D. (2016). Mental imagery of speech implicates two mechanisms of perceptual reactivation. *Cortex* 77 1–12. 10.1016/j.cortex.2016.01.002 26889603 PMC5357080

[B70] Tolomeo S. Christmas D. Jentzsch I. Johnston B. Sprengelmeyer R. Matthews K. (2016). A causal role for the anterior mid-cingulate cortex in negative affect and cognitive control. *Brain* 139(Pt 6) 1844–1854. 10.1093/brain/aww069 27190027

[B71] Uddin L. Q. Kinnison J. Pessoa L. Anderson M. L. (2014). Beyond the tripartite cognition-emotion-interoception model of the human insular cortex. *J. Cogn. Neurosci.* 26 16–27. 10.1162/jocn_a_00462 23937691 PMC4074004

[B72] Vicentini J. E. Weiler M. Almeida S. R. M. de Campos B. M. Valler L. Li L. M. (2017). Depression and anxiety symptoms are associated to disruption of default mode network in subacute ischemic stroke. *Brain Imaging Behav.* 11 1571–1580. 10.1007/s11682-016-9605-7 27743373

[B73] Wang S. Yang M. Du S. Yang J. Liu B. Gorriz J. M. (2016). Wavelet entropy and directed acyclic graph support vector machine for detection of patients with unilateral hearing loss in MRI scanning. *Front. Comput. Neurosci.* 10:106. 10.3389/fncom.2016.00106 27807415 PMC5069288

[B74] Wang Z. Aguirre G. K. Rao H. Wang J. Fernandez-Seara M. A. Childress A. R. (2008). Empirical optimization of ASL data analysis using an ASL data processing toolbox: ASLtbx. *Magnet. Reson. Imaging* 26 261–269. 10.1016/j.mri.2007.07.003 17826940 PMC2268990

[B75] Williams D. S. Detre J. A. Leigh J. S. Koretsky A. P. (1992). Magnetic-resonance-imaging of perfusion using spin inversion of arterial water. *Proc. Natl. Acad. Sci. U.S.A.* 89 212–216. 10.1073/pnas.89.1.212 1729691 PMC48206

[B76] Xekardaki A. Rodriguez C. Montandon M. L. Toma S. Tombeur E. Herrmann F. R. (2015). Arterial spin labeling may contribute to the prediction of cognitive deterioration in healthy elderly individuals. *Radiology* 274 490–499. 10.1148/radiol.14140680 25291458

[B77] Yang M. Chen H. J. Liu B. Huang Z. C. Feng Y. Li J. (2014). Brain structural and functional alterations in patients with unilateral hearing loss. *Hear Res.* 316 37–43. 10.1016/j.heares.2014.07.006 25093284

[B78] Zamorano A. M. Cifre I. Montoya P. Riquelme I. Kleber B. (2017). Insula-based networks in professional musicians: Evidence for increased functional connectivity during resting state fMRI. *Hum. Brain Mapp.* 38 4834–4849. 10.1002/hbm.23682 28737256 PMC6866802

[B79] Zhang Y. Nayak D. R. Yang M. Yuan T. F. Liu B. Lu H. (2017a). Detection of unilateral hearing loss by stationary wavelet entropy. *CNS Neurol. Disord. Drug Targets* 16 122–128. 10.2174/1871527315666161026115046 27784224

[B80] Zhang Y. Yang Y. Bian R. Yin Y. Hou Z. Yue Y. (2017b). Group cognitive behavior therapy reversed insula subregions functional connectivity in asthmatic patients. *Front. Aging Neurosci.* 9:105. 10.3389/fnagi.2017.00105 28458637 PMC5394595

[B81] Zhang Y. Yang Y. Wang Z. Bian R. Jiang W. Yin Y. (2018). Altered regional cerebral blood flow of right cerebellum posterior lobe in asthmatic patients with or without depressive symptoms. *Front. Psychiatry* 9:225. 10.3389/fpsyt.2018.00225 29892237 PMC5985698

[B82] Zhou S. Y. Suzuki M. Hagino H. Takahashi T. Kawasaki Y. Matsui M. (2005). Volumetric analysis of sulci/gyri-defined in vivo frontal lobe regions in schizophrenia: Precentral gyrus, cingulate gyrus, and prefrontal region. *Psychiatry Res.* 139 127–139. 10.1016/j.pscychresns.2005.05.005 15967647

[B83] Zung W. W. (1971). A rating instrument for anxiety disorders. *Psychosomatics* 12 371–379. 10.1016/S0033-3182(71)71479-0 5172928

